# An miRNA signature associated with tumor mutation burden in endometrial cancer

**DOI:** 10.1042/BSR20203398

**Published:** 2020-11-13

**Authors:** Hongyu Zhou, Lihua Chen, Mei Qin, Yajie Lei, Tianjiao Li, Haoran Li, Xi Cheng

**Affiliations:** 1 Department of Gynecologic Oncology, Fudan University Shanghai Cancer Center, Shanghai, China; 2 Department of Oncology, Shanghai Medical College, Fudan University, Shanghai, China; 3 Cancer Institute, Fudan University Shanghai Cancer Center, Shanghai, China; 4 Department of Gynecology and Obstetrics, Zhuzhou Hospital, Xiangya School of Medicine, Central South University, Zhu Zhou, Hunan, China; 5 Department of Pancreatic Surgery, Fudan University Shanghai Cancer Center, Shanghai, China

**Keywords:** endometrial cancer, immunotherapy, miRNA, TCGA, tumor mutation burden

## Abstract

Tumor mutation burden (TMB) is an essential biomarker to predict immunotherapy response. TMB measurement was mainly evaluated by whole-exome sequencing (WES), which was costly and difficult to be widely applied. In the present study, we aimed to establish and validate a miRNA signature to predict TMB level in endometrial cancer using The Cancer Genome Atlas (TCGA) database. MiRNA expression and somatic mutation profiles of uterine corpus endometrial carcinoma (UCEC) were downloaded from TCGA database. Total 518 patients with UCEC were randomly classified into training set (*n*=311) and validation set (*n*=207). Thirty-five differentially expressed miRNAs between high-TMB and low-TMB group were identified in training set. Least absolute shrinkage and selection operator (LASSO) method was performed to select out 26 miRNAs to establish the optimal signature. The accuracy of the miRNA signature for predicting TMB level was 0.833 for training set, 0.749 for validation set and 0.799 for total set. Moreover, the miRNA signature had significant correlation with immune checkpoints related genes (PD-1, PD-L1, CTLA-4) and mismatch repair related genes (BRCA1, BRCA2, *MLH1, MSH6*) expression. In conclusion, this miRNA signature could predict TMB level in endometrial cancer and might have some merits in providing guidance for immunotherapy in endometrial cancer.

## Introduction

Endometrial cancer was the fourth women cancer worldwide and the commonest gynecological malignancy in developed countries [1,2]. There were 61,880 new cases and 12,160 deaths of uterine corpus carcinoma in 2019 estimated by American Cancer Society [1]. Generally, majority (67%) of patients were diagnosed in early stage with localized diseases and approximately 30% of patients had regional/distant diseases in late stage [1]. Early-stage patients could achieve favorable outcomes with 5-year overall survival rate of 80–95%, but patients with advanced stage had decreased survival with 5-year survival rate of 68% and 17% for stage III and stage IV, respectively [3,4]. Localized patients could benefit from radical surgery; however, for metastatic/recurrent patients, current therapeutic strategies presented limited survival benefit [5,6].

In recent years, immunotherapy, including anti-programmed death‐1 (PD-1)/anti-programmed death‐ligand‐1 (PD-L1) inhibitors and anti-cytotoxic T-lymphocyte antigen 4 (CLAT-4) inhibitors, had greatly implemented therapeutic advancements in various types of recurrent/metastatic cancers [7–9]. Pembrolizumab (a PD-1 immune checkpoint inhibitor) was approved by Food and Drug Administration (FDA), manifested good response in mismatch repair deficient (dMMR)/microsatellite instability-high (MSI-H) solid tumors including colon cancer and endometrial cancer [10,11]. In a phase 2 clinical trial, approximately 20% (3/15) of patients with dMMR/MSI-H endometrial cancer had a complete response and 33% (5/15) had a partial response after anti-PD-1 treatment [12].

Tumor mutation burden (TMB), an essential biomarker for immunotherapy response, was defined as whole number of gene variants [13]. High TMB manifested a large count of mutated genes, which encoded aberrant tumor neoantigen [14]. Higher TMB suggested better response to immunotherapy in various tumor types [13,15]. TMB assessment was largely from whole exome sequencing, which needed sufficient tumor samples and was extremely expensive to be widely implemented. Therefore, a costless and convenient tool to predicting TMB level was in urgent needed. Mutated genes underwent transcriptional and post-transcriptional modifications to generate abnormal oncoproteins, which triggered anti-cancer immune response. MicroRNAs (miRNAs) were viewed as crucial regulators in post-translational gene modifications [16].

MiRNAs were small, non-coding RNA molecules, approximately 20–22 nucleotides in length, participating in post-transcriptional modification process and degradation of targeted messenger RNAs, which was involved in tumor proliferation/tumor suppression [16]. In the past few years, several researches had revealed that miRNAs could be candidate biomarkers for carcinogenesis, diagnosis/differential diagnosis and prognosis of gynecological cancers [17–20]. Regarding endometrial cancer, miRNAs appeared associated with initiating lesions and worse clinical outcomes such as positive lymph node status, lymph-vascular space invasion, shorter overall survival [21,22]. However, the role of miRNAs in personalized cancer treatment has been not fully elucidated yet. A study conducted by Peng et al. [23] revealed that three miRNAs (hsa-miR-320d, hsa-miR-320c, hsa-miR-320b) might predict immunotherapy response in non-small cell lung cancer.

MiRNAs were crucial in post-transcriptional modifications of gene expression regulation. Theoretically, mutated genes might be modified by miRNAs, encoding loads of neoantigens. However, the association between miRNAs and TMB level was not investigated before. In the present study, we hypothesized that TMB level might affect miRNAs expression and differentially expressed miRNAs might predict TMB level. In the present study, we tried to establish and validate a miRNA signature to predicting TMB level in endometrial cancer from The Cancer Genome Atlas (TCGA) database.

## Methods

### Data source and cases grouping

First, transcriptome data and gene expression data of uterine corpus endometrial carcinoma (UCEC) including 25 normal tissues and 552 tumor samples were downloaded from TCGA database via GDC portal (https://portal.gdc.cancer.gov/). Second, we obtained somatic mutation profiles of 530 UCEC samples from ‘Masked Somatic Mutation’ category in TCGA database (https://cancergenome.nih.gov/), which included four subtypes of mutation profiles by four different software process. We selected somatic mutation profiles based on ‘MuTect2 Variant Aggregation and Masking’ process for subsequent analysis. Third, isoform expression data of UCEC (22 matched normal tissues and 546 tumor samples) were acquired from TCGA database.

Clinical information of 518 patients with UCEC on age at diagnosis, race, ethnicity, menopause status, histological type, tumor grade, clinical stage, survival time, survival status was collected. Final 518 patients with miRNA expression data and somatic mutation data were used for further analysis in the present study. Then, total 518 patients were randomly assigned into training set (60%) and validation set (40%) by R package ‘caret’. The flow chart of study design was shown in Supplementary Figure S1.

### Differentially expressed miRNA in two TMB groups in the training set

TMB, defined as total number of somatic gene coding errors, could be calculated as (whole counts of gene variants)/(whole length of exons) [14]. We calculated TMB of each samples via Perl script (https://www.perl.org/) and divided all patients into the low TMB group and the high TMB group by the median TMB. Then, we screened for all miRNAs and selected out differentially expressed miRNAs in two TMB groups with fold change ≥1.5 and FDR<0.01 by R package ‘limma’. All differentially expressed miRNAs were presented in heatmap by R ‘pheatmap’ package.

### Construction and validation of TMB-related miRNA signature by LASSO

The least absolute shrinkage and selection operator (LASSO) method with a powerful predictive value and a low correlation between each other to prevent overfitting was applied to select optimal features for the high-dimensional data [24]. LASSO regression method was performed to establish optimal miRNA signature to predicting TMB level in training set by R package ‘glmnet’. Each miRNAs in this signature had their regression coefficients (β) for predicting TMB and the classifier index could be calculated as: index = (expression of miRNA_1_)*β_1_ + (expression of miRNA_2_)*β_2_ + (expression of miRNA_3_)*β_3_……..+ (expression of miRNA_n_)*β_n_. Receiver operating curve (ROC) analysis was performed to verify the accuracy of the miRNA signature in total set, training set and validation set. Principal component analysis (PCA) was used prior to LASSO method to present all differentially expressed miRNA for predicting TMB level and PCA was used after LASSO method to present optimal miRNAs in the signature for predicting TMB level. All samples were presented in two-dimensional plots by PCA.

### Functional analysis

Kyoto Encyclopedia of Genes and Genomes (KEGG) and Gene Ontology (GO) analysis were performed in differentially expressed miRNAs in the signature between high TMB group and low TMB group via online DIANA-mirPath software (version:3.0) derived from TarBase 7.0 (http://snf-515788.vm.okeanos.grnet.gr/) with *P* value <0.01 [25]. Then, top 20 significant biological pathways of KEGG and biological process (BP) component of GO analysis were visualized in bubble plot by R package ‘ggplot2’. Moreover, we investigated the targets of all differentially expressed miRNAs in this signature via three miRNA target-predicting database (miRDB, TargetScan and miRTarBase database) [26–28]. All predicted targets in these three database were presented in Venn diagrams by R package ‘VennDiagram’.

### Correlation of miRNA-based signature with TMB and gene expression

We extracted gene expression of each samples from TCGA database and calculated TMB level and signature index of each samples. Then we investigated the association between the miRNA signature and TMB level and several genes expression including mismatch repair (MMR) related genes (BRCA1, BRCA2, MLH1, MSH2, MSH6, PSM2) and immune checkpoints related genes (PD-1, PD-L1, CTLA-4) by Spearman method.

### Statistical analysis

All statistical analysis was conducted by R software (version 4.0.0) for windows. The statistical significance of all clinicopathological characteristics in training set and validation set was tested by Chi-Square test. The significance with the continuous variables such as age at diagnosis and TMB were assessed by nonparametric tests (e.g., rank sum tests). Statistical significance was set by *P*<0.05.

## Results

### Differentially expressed miRNA in two TMB groups

**Figure 1:**
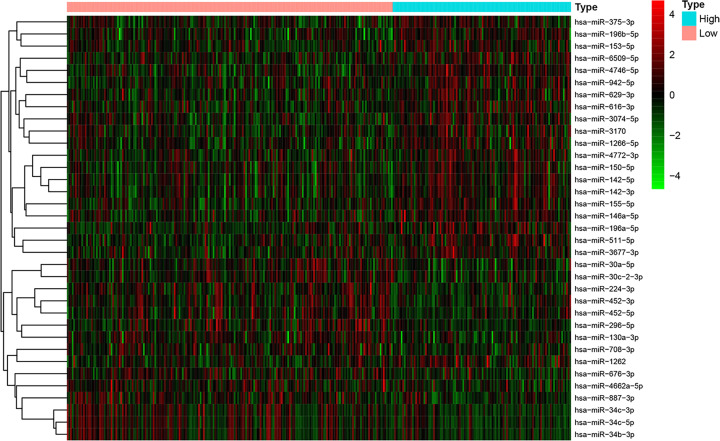
The heatmap of 35 differentially expressed miRNAs between high-TMB group and low-TMB group Each columns represented each samples. The colors in the heatmaps from green to red represented miRNA expression level from low to high. The red dots in the heatmap represented up-regulation, the green dots represented down-regulation and black dots represented miRNAs without differential expression. At the top of the heatmap, the light blue color represented samples with high TMB level and the light red color represented samples with low TMB level.

Total 518 patients with UCEC from TCGA database were randomized into the training set (*n*=311) and validation set (*n*=207) (Supplementary Figure S1). Demographics and clinicopathological characteristics of these two cohorts presented no significant difference, shown in Table 1. Then 35 differentially expressed miRNAs in these two TMB groups were selected out by FC ≥1.5 and *P* value <0.01. All differentially expressed miRNAs in each samples were presented in heatmap plot (Figure 1). Among these 35 differentially expressed miRNAs, 21 miRNAs were up-regulated and 14 miRNAs were down-regulated in high TMB group (Supplementary Table S1).

**Table 1:** Demographics and clinicopathological characteristics of 518 patients with UCEC from the TCGA database

Clinical variables	Total set number (%)	Training set number (%)	Validating set number (%)	*P* value
Age at diagnosis				0.428
£64 y	278(53.67%)	162(52.09%)	116(56.04%)	
>64 y	240(46.33%)	149(47.91%)	91(43.96%)	
Ethnicity				0.162
Hispanic/Latino	14(2.7%)	8(2.57%)	6(2.9%)	
Not Hispanic/Latino	362(69.88%)	227(72.99%)	135(65.22%)	
Unknown	142(27.41%)	76(24.44%)	66(31.88%)	
Race				0.972
African	105(20.27%)	62(19.94%)	43(20.77%)	
Caucasian	353(68.15%)	213(68.49%)	140(67.63%)	
Other	60(11.58%)	36(11.58%)	24(11.59%)	
Menopause status				0.997
Pre	32(6.18%)	19(6.11%)	13(6.28%)	
Post	441(85.14%)	265(85.21%)	176(85.02%)	
Unknown	45(8.69%)	27(8.68%)	18(8.7%)	
Histological type				0.297
EAC	384(74.13%)	223(71.7%)	161(77.78%)	
SAC	8(1.54%)	5(1.61%)	3(1.45%)	
Other	126(24.32%)	83(26.69%)	43(20.77%)	
Grade				0.858
Grade I/II	214(41.31%)	127(40.84%)	87(42.03%)	
Grade III/IV	304(58.69%)	184(59.16%)	120(57.97%)	
Clinical stage				0.431
Stage I-II	325(62.74%)	202(64.95%)	123(59.42%)	
Stage III-IV	48(9.27%)	24(7.72%)	24(11.59%)	

Abbreviations: EAC, endometrioid adenocarcinoma; SAC, serous adenocarcinoma.

### Construction and validation of miRNA signature

**Figure 2:**
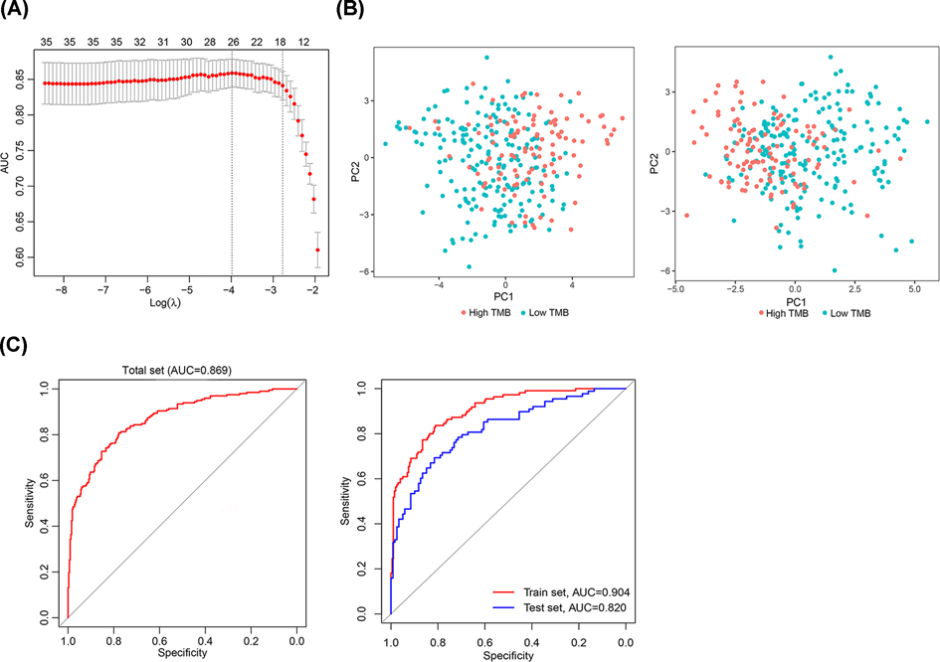
Construction and validation of miRNA signature (**A**) LASSO method to select out optimal miRNAs for the signature. 10-fold cross-validation for tuning parameter selection was used in the LASSO model. (**B**) PCA analysis for all differentially expressed miRNAs (left) and 25 miRNAs in the signature (right) in the training set. (**C**) ROC analysis for the miRNA signature in the total set, the training set and the validation set.

For establishing a TMB-related miRNA signature in UCEC, two-class LASSO logistic regression model was conducted in the training set using group-wise classifications with 10-fold cross-validation and ‘AUC’ measure type (Figure 2A). Twenty-six miRNAs (hsa-miR-887-3p, hsa-miR-511-5p, hsa-miR-3677-3p, hsa-miR-4746-5p, hsa-miR-196a-5p, hsa-miR-3074-5p, hsa-miR-708-3p, hsa-miR-296-5p, hsa-miR-224-3p, hsa-miR-676-3p, hsa-miR-196b-5p, hsa-miR-153-5p, hsa-miR-142-5p, hsa-miR-1266-5p, hsa-miR-616-3p, hsa-miR-155-5p, hsa-miR-452-3p, hsa-miR-130a-3p, hsa-miR-1262, hsa-miR-452-5p, hsa-miR-375-3p, hsa-miR-30a-5p, hsa-miR-34b-3p, hsa-miR-34c-3p, hsa-miR-30c-2-3p, hsa-miR-4662a-5p) were selected out as optimal features with non-zero regression coefficients (λ) and incorporated into this signature. By PCA analysis prior to and after LASSO method, we verified that this optimal miRNA signature including these 26 miRNAs had better discrimination and predictability for TMB level than that including all 35 differentially expressed miRNAs in the training set (Figure 2B).

Each miRNAs had their coefficients to predicting TMB level. As shown in Supplementary Table S2, the index of the signature could be calculated as follows: index = (-4.72) + hsa-miR-887-3p*(-0.08) + hsa-miR-511-5p*(0.18) + hsa-miR-3677-3p*(0.03) + hsa-miR-4746-5p * (0.25) + hsa-miR-196a-5p *(0.04) + hsa-miR-3074-5p * (0.16) + hsa-miR-708-3p* (-0.08) + hsa-miR-296-5p* (-0.21) + hsa-miR-224-3p *(-0.23) + hsa-miR-676-3p *(-0.35) + hsa-miR-196b-5p* (0.24) + hsa-miR-153-5p*(0.08) + hsa-miR-142-5p*(0.03) + hsa-miR-1266-5p *(0.09) + hsa-miR-616-3p*(0.14) + hsa-miR-155-5p*(0.27) + hsa-miR-452-3p*(-0.02) + hsa-miR-130a-3p*(-0.17) + hsa-miR-1262*(0.33) + hsa-miR-452-5p*(-0.07) + hsa-miR-375-3p*(0.03) + hsa-miR-30a-5p(-0.02) + hsa-miR-34b-3p*(-0.12) + hsa-miR-34c-3p*(-0.12) + hsa-miR-30c-2-3p*(-0.01) + hsa-miR-4662a-5p*(-0.05). ROC analysis was conducted to investigate the accuracy of the signature. The accuracy value was 0.833 for training set, 0.749 for validation set and 0.799 for total set. Area under the curves (AUCs) for total set, training set and validation set were 0.869, 0.904 and 0.820, respectively (Figure 2C). Other model indexes as sensitivity (Se), specificity (Sp), positive predictive value and negative predictive value were shown in Table 2.

**Table 2:** Model indexes for the miRNA signature in the total set, the training set, and validation set

Set	Se	Sp	PPV	NPV	Accuracy	AUC
Training set	0.682	0.915	0.815	0.840	0.833	0.904
Validating set	0.568	0.882	0.781	0.734	0.749	0.820
Total set	0.631	0.903	0.801	0.798	0.799	0.869

Se = sensitivity; Sp = specificity, PPV = positive predictive value; NPV = negative predictive value; AUC = area under the curve.

### Function analysis of miRNAs in the signature

**Figure 3:**
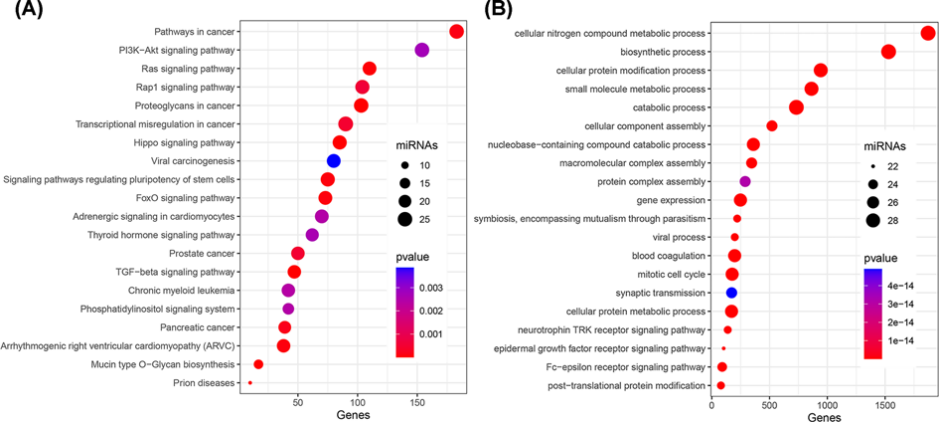
KEGG (A) and GO (B) enrichment analysis of 25 differentially expressed miRNAs in the signature Top 20 significant pathways were shown in bubble plots.

To investigate the functional role of 26 miRNAs in the signature, we performed KEGG and GO analysis via DIANA-mirPath software. We discovered that these miRNAs were mostly enriched in carcinogenesis process and activated tumor proliferation pathways (Ras pathway, PI3K-Akt pathway and Rap1 pathway) by KEGG analysis (Figure 3A). For BP process in GO analysis, most miRNAs were enriched in cellular metabolism and protein modifications process (Figure 3B and Supplementary Table S3). Via three targets predicting database (miRDB, TargetScan and miRTarBase database), we discovered that MMR-related genes (BRCA1, BRCA2, MLH1, MSH2, MSH6, PMS2) and immune checkpoints related genes (PD-1, PD-L1 and CTLA-4) were possible targets for 26 miRNAs in the signature (Supplementary Table S4). All predicted targets of 26 miRNAs were shown in Venn diagram (Supplementary Figure S2).

### Association between the miRNA signature and TMB

**Figure 4:**
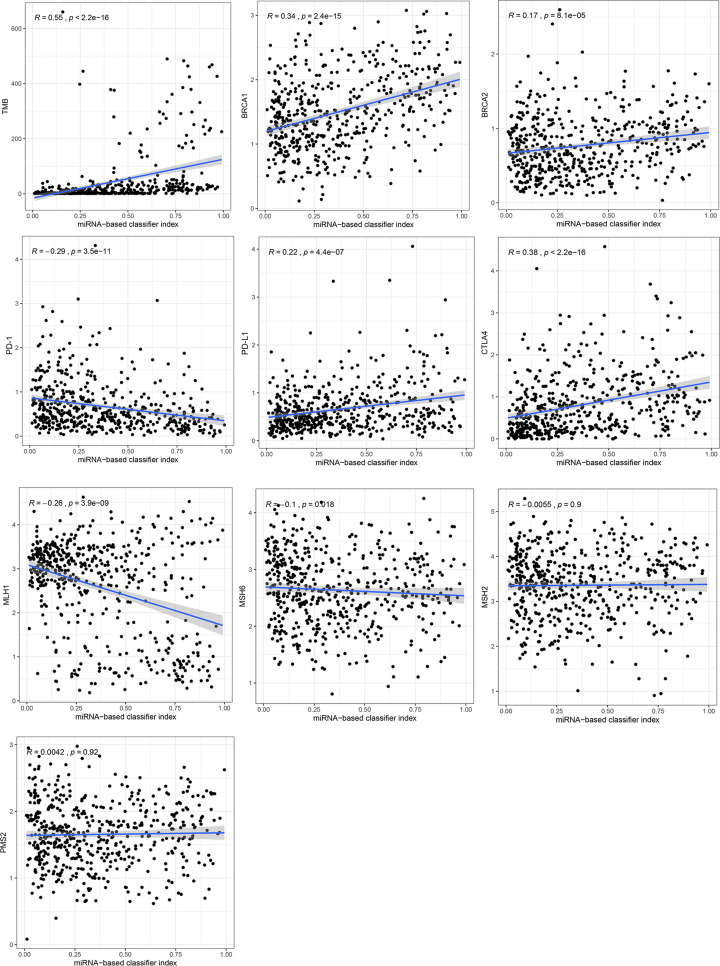
Correlation analysis of the miRNA signature with TMB level and several genes expression

Furthermore, we investigated the correlation of the miRNA signature with TMB level and several genes expression (Figure 4). We discovered that the miRNA signature had significantly positive correlation with TMB level (*R* = 0.55, *P*<0.001), BRCA1 expression (*R* = 0.34, *P*<0.001), BRCA2 expression (*R* = 0.17, *P*<0.001), PD-L1 expression (*R* = 0.22, *P*<0.001), CTLA4 expression (*R* = 0.38, *P*<0.001), but had significantly negative correlation with PD-1 expression (*R* = -0.29, *P*<0.001), MLH1 expression (*R* = -0.26, *P*<0.001), MSH6 expression (*R* = -0.1, *P*=0.018). However, MSH2 expression and PMS2 expression had no significantly correlation with the signature with *P* value >0.05.

## Discussion

Immunotherapy had been initiated in advanced/recurrent endometrial cancer manifesting good response. In the Keynote 028 clinical trial (Pembrolizumab) enrolling 24 patients with PD-L1 positive endometrial cancer, the overall response rate was 13% and 6-month progression-free survival and overall survival were 19%, 68.8%, respectively [10]. Despite of PD-L1 expression, dMMR/MSI-H endometrial cancers also well responded to immunotherapy [12]. TMB level was viewed as another indicator for immunotherapy response and higher TMB was associated with longer overall survival after immunotherapy across multiple cancer types [13,29]. However, the golden standard for TMB measurement was whole-exome sequencing, which was costly and difficult for many institutions to put into use. Therefore, alternative methods for predicting TMB level were needed. In the present study, our group was the first to establish and validate a TMB-related miRNA signature in endometrial cancer, and we discovered that this signature had good predictability for TMB level and had potential to be an alternative tool to estimate TMB level in endometrial cancer.

To investigate functional role of these differentially expressed miRNAs in the signature, we performed KEGG and GO analysis to find out they were mostly enriched in carcinogenesis related process and tumor proliferation pathway (Ras, PI3K-Akt, Rap1 pathway). Furthermore, we predicted possible targeted genes for these miRNAs, and surprisingly discovered that immune checkpoints related genes (PD-1, PD-L1, CTLA4) and MMR-related genes (BRCA1, BRCA2, MLH1, MSH2, MSH6, PSM2) were potential targets for the signature. Above these findings, we explored the correlation between the miRNA signature and these genes expression, we discovered that this signature was positively correlated to the expression of PD-L1, CTLA4 but negatively correlated to PD-1 expression. Despite immune checkpoints related genes, MMR-related genes (BRCA1, BRCA2, MLH1, MSH6) were also proven association with the signature. In fact, Pembrolizumab was recommended to all MSI-H solid tumors and 78% had responses that lasting for at least 6 months; however, most endometrial cancers were microsatellite stable (MSS) [2]. MSS endometrial cancers might focus more on targeted therapy such as anti-angiogenic agents and poly (ADP-ribose) polymerase (PARP) inhibitors. Based on our findings that this miRNA-based signature had correlation with MMR-related genes, it might have some merits in providing guidance for PARP inhibitors against MMR deficiency in endometrial cancer.

Our study innovatively established and validated a miRNA signature to predict TMB level, which might provide evidence for the predicting value of miRNA on immunotherapy response. Meanwhile, this signature could predict the expression of immune checkpoints related genes and MMR-related genes, which will promote further exploration of immunotherapy and targeted therapy in endometrial cancer. However, this study was limited to a database study, the efficiency and accuracy of the miRNAs signature needed more clinical investigations to verify.

## Conclusions

We demonstrated a miRNA signature was a useful tool to predict TMB level in endometrial cancer and had a correlation with expression of immune checkpoints genes and MMR-related genes.

## Supplementary Material

Supplementary Figure S1-S2

Supplementary Tables S1-S4

## Data Availability

Somatic mutation profiles and miRNA expression data of UCEC were available in TCGA database (https://cancergenome.nih.gov/). The biological pathways of differentially expressed miRNAs were analyzed by DIANA-mirPath software (version:3.0) (http://snf-515788.vm.okeanos.grnet.gr/).

## Abbreviations

AUC: area under the curve
CTLA4: cytotoxic T-lymphocyte antigen 4
GO: gene ontology
KEGG: Kyoto Encyclopedia of Genes and Genomes
LASSO: least absolute shrinkage and selection operator
MMR: mismatch repair
PCA: principal component analysis
PD-1: programmed death‐1
PD-L1: programmed death‐ligand‐1
ROC: receiver operating curve
TCGA: the cancer genome atlas
TMB: tumor mutation burden
UCEC: uterine corpus endometrial carcinoma
