# Supplementary material for: An miRNA signature associated with tumor mutation burden in endometrial cancer
Source: Biosci Rep. 2020 Nov 13;40(11):BSR20203398. doi: 10.1042/BSR20203398 (PMC7670578; doi:10.1042/BSR20203398)
Supplement: Supplementary Figure S1-S2 [file BSR-2020-3398_supp.pdf]

Supplementary Figure 1.

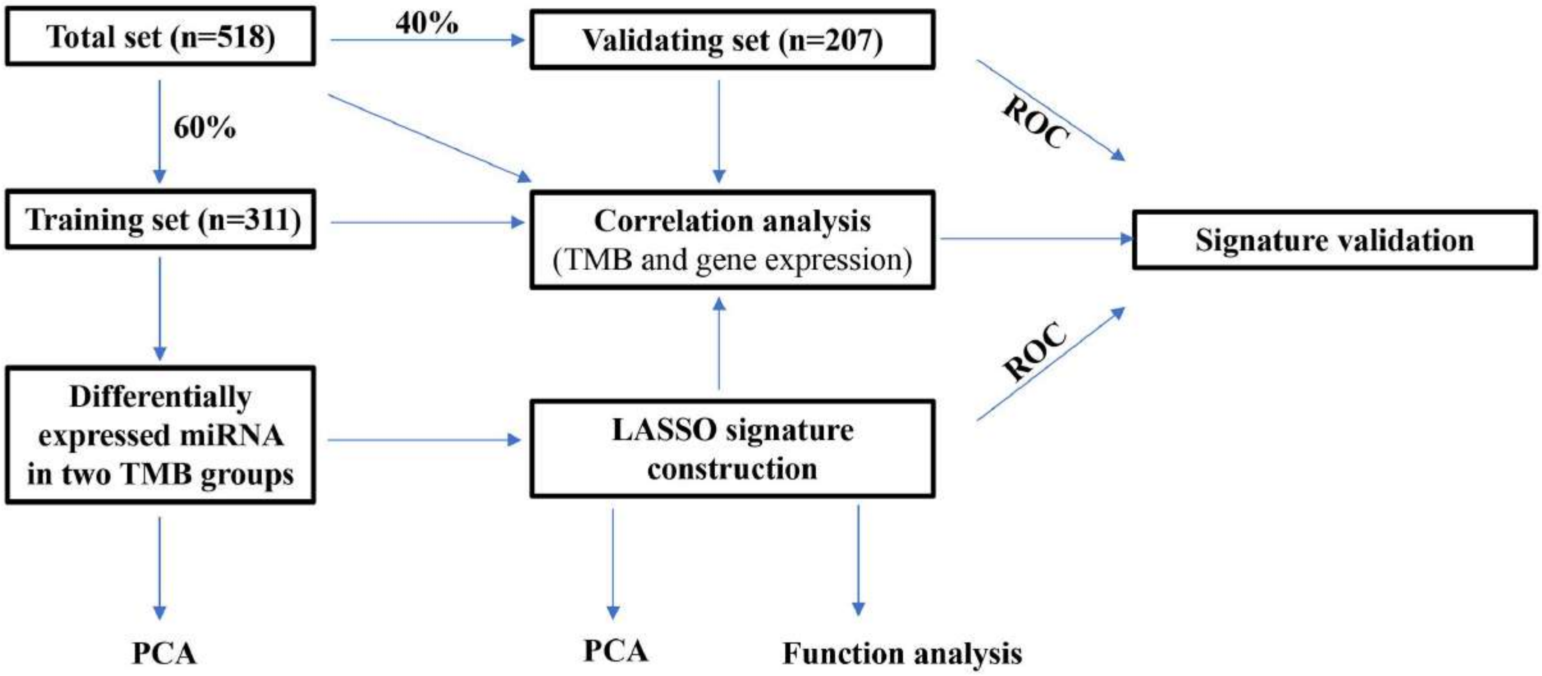

Supplementary Figure 1. Flow chart of the whole study design.

Supplementary Figure 2.

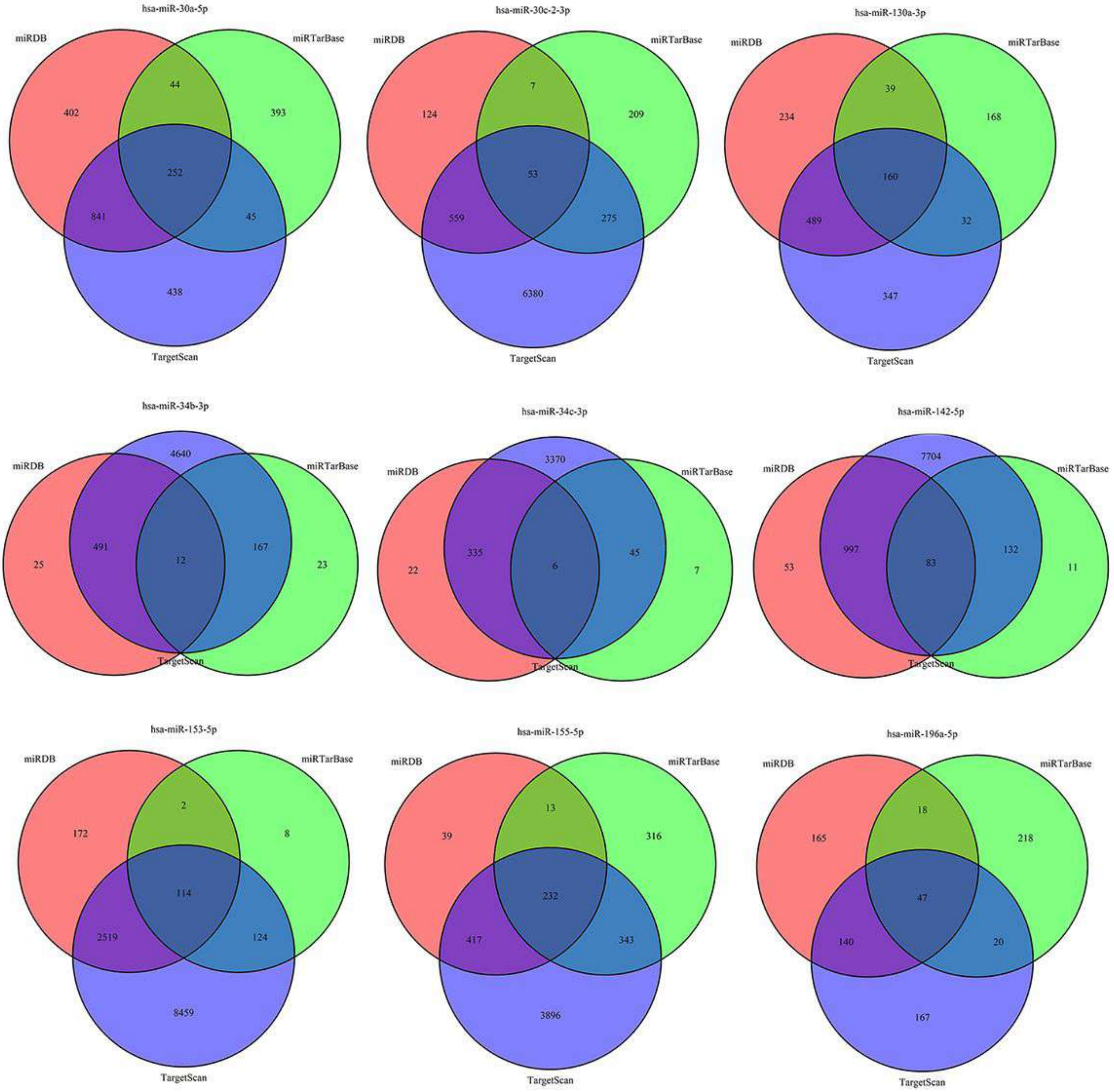

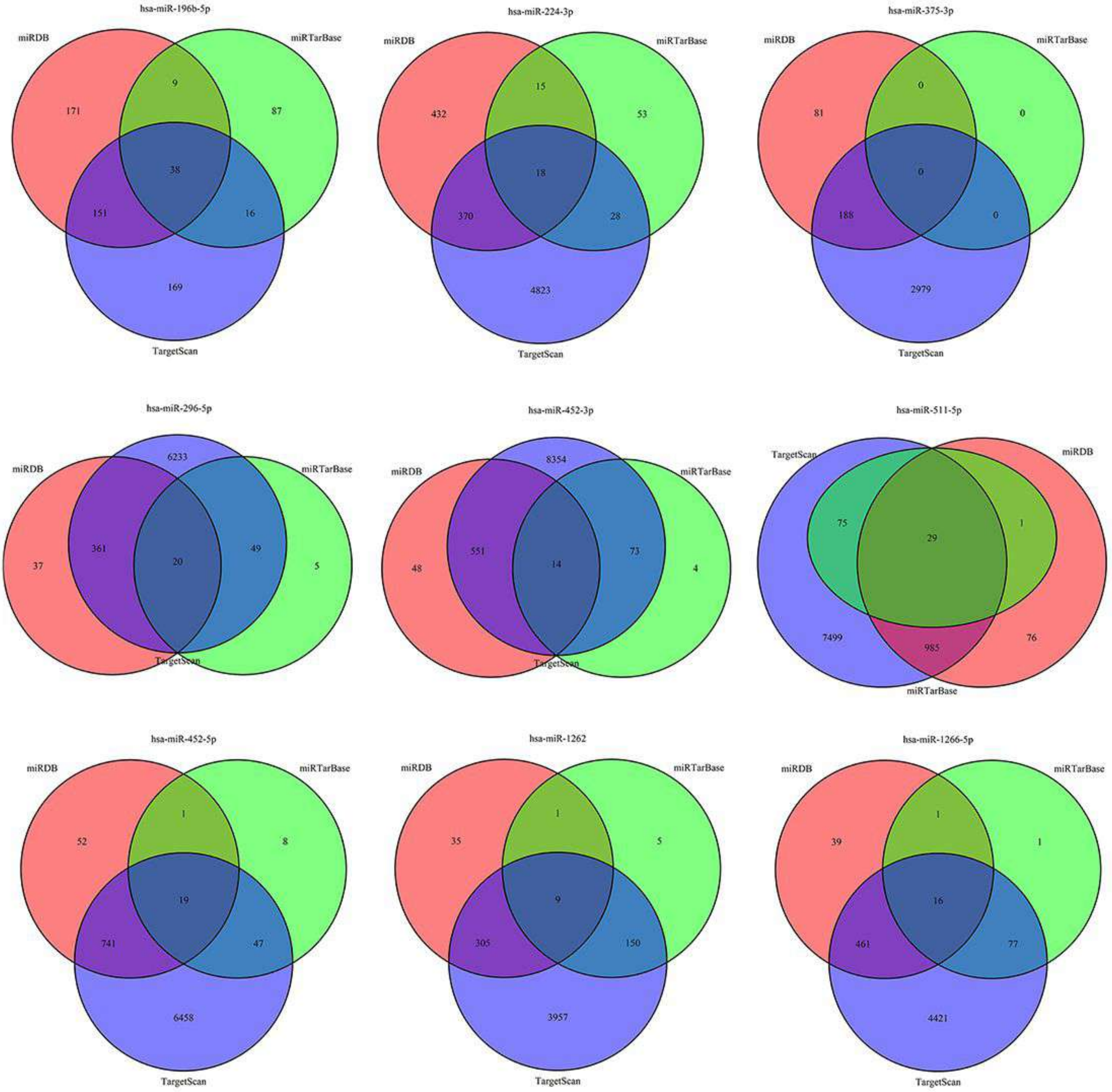

hsa-miR-196b-5p
miRDB
miRTarBase
TargetScan
171
9
87
38
151
16
169
hsa-miR-224-3p
miRDB
miRTarBase
TargetScan
432
15
53
18
370
28
4823
hsa-miR-375-3p
miRDB
miRTarBase
TargetScan
81
0
0
0
188
0
2979
hsa-miR-296-5p
miRDB
miRTarBase
TargetScan
6233
37
361
20
49
5
hsa-miR-452-3p
miRDB
miRTarBase
TargetScan
8354
48
551
14
73
4
hsa-miR-511-5p
TargetScan
miRDB
miRTarBase
75
29
1
7499
985
76
hsa-miR-452-5p
miRDB
miRTarBase
TargetScan
52
1
8
19
741
47
6458
hsa-miR-1262
miRDB
miRTarBase
TargetScan
35
1
5
9
305
150
3957
hsa-miR-1266-5p
miRDB
miRTarBase
TargetScan
39
1
1
16
461
77
4421

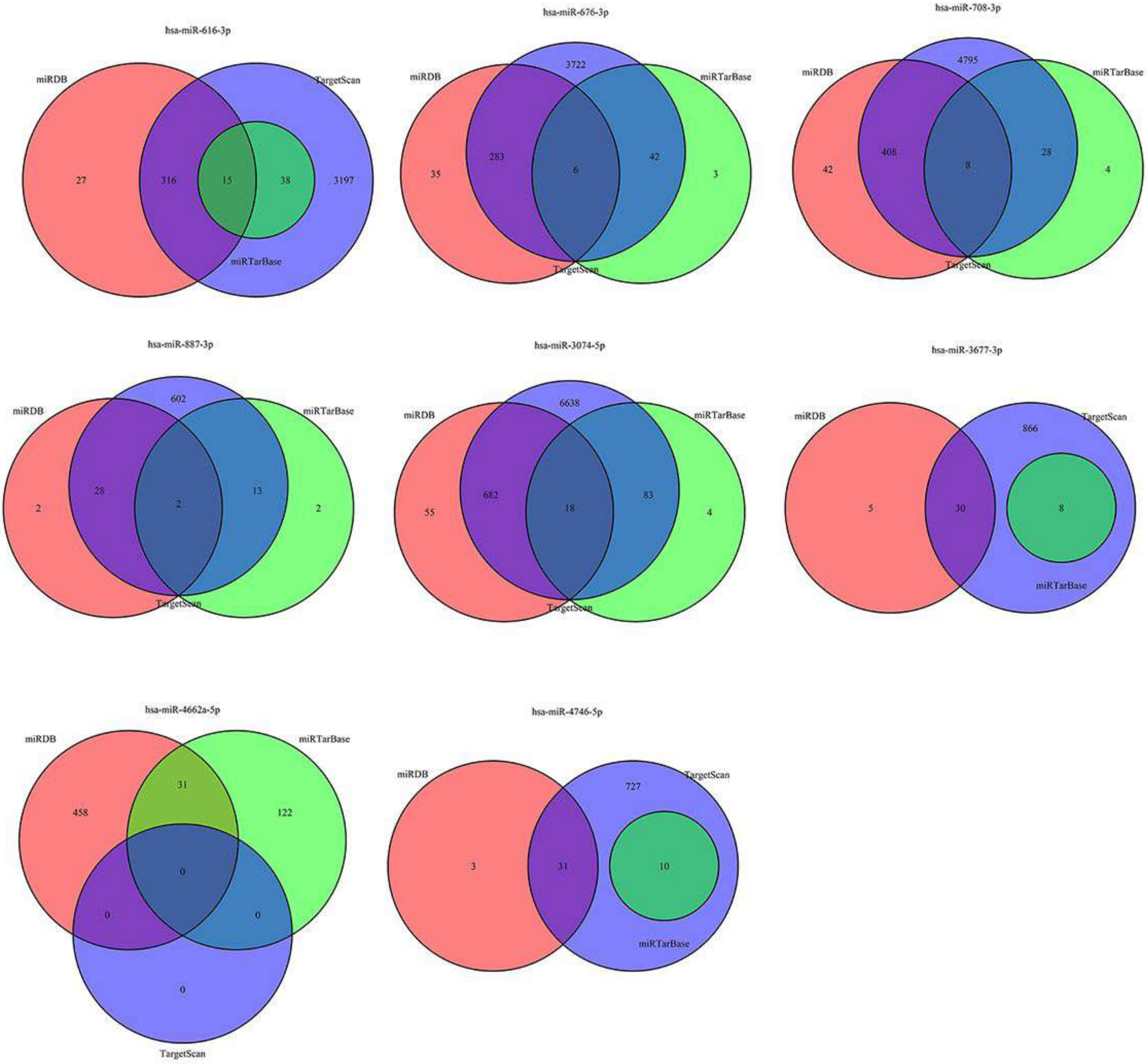
hsa-miR-616-3p
miRDB
TargetScan
miRTarBase
27
316
15
38
3197
hsa-miR-676-3p
miRDB
miRTarBase
TargetScan
3722
35
283
6
42
3
hsa-miR-708-3p
miRDB
miRTarBase
TargetScan
4795
42
408
8
28
4
hsa-miR-887-3p
miRDB
miRTarBase
TargetScan
602
2
28
2
13
2
hsa-miR-3074-5p
miRDB
miRTarBase
TargetScan
6638
55
682
18
83
4
hsa-miR-3677-3p
miRDB
TargetScan
miRTarBase
866
5
30
8
hsa-miR-4662a-5p
miRDB
miRTarBase
TargetScan
458
31
122
0
0
0
0
hsa-miR-4746-5p
miRDB
TargetScan
miRTarBase
727
3
31
10

Supplementary Figure 2. The Venn diagrams of predicted targets for 25 miRNAs in the signature.
